# Field-deployable, rapid diagnostic testing of saliva for SARS-CoV-2

**DOI:** 10.1038/s41598-021-84792-8

**Published:** 2021-03-09

**Authors:** Shan Wei, Hemant Suryawanshi, Alexandre Djandji, Esther Kohl, Stephanie Morgan, Eldad A. Hod, Susan Whittier, Kevin Roth, Raymond Yeh, Juan Carlos Alejaldre, Elaine Fleck, Stephen Ferrara, Daniel Hercz, David Andrews, Lilly Lee, Kristopher A. Hendershot, Joshua Goldstein, Yousin Suh, Mahesh Mansukhani, Zev Williams

**Affiliations:** 1 Columbia University Irving Medical Center, New York, NY USA; 2 New York Presbyterian Hospital, New York, NY USA; 3 Jackson Memorial Hospital, Miami, FL USA

**Keywords:** DNA, Infectious-disease diagnostics, SARS-CoV-2, Assay systems

## Abstract

To safely re-open economies and prevent future outbreaks, rapid, frequent, point-of-need, SARS-CoV-2 diagnostic testing is necessary. However, existing field-deployable COVID-19 testing methods require the use of uncomfortable swabs and trained providers in PPE, while saliva-based methods must be transported to high complexity laboratories for testing. Here, we report the development and clinical validation of High-Performance Loop-mediated isothermal Amplification (HP-LAMP), a rapid, saliva-based, SARS-CoV-2 test with a limit of detection of 1.4 copies of virus per µl of saliva and a sensitivity and specificity with clinical samples of > 96%, on par with traditional RT-PCR based methods using swabs, but can deliver results using only a single fluid transfer step and simple heat block. Testing of 120 patient samples in 40 pools comprised of 5 patient samples each with either all negative or a single positive patient sample was 100% accurate. Thus, HP-LAMP may enable rapid and accurate results in the field using saliva, without need of a high-complexity laboratory.

## Introduction

Frequent, rapid, sensitive, and accurate COVID-19 testing that can be scaled and deployed in the field is critical for controlling the ongoing pandemic and preventing future outbreaks^1–3^. However, existing methods either use nasal/nasopharyngeal swabs, which require the use and exposure of trained personnel and personal protective equipment (PPE) and are less conducive to frequent testing in the general population, or use saliva but must be transported to high complexity laboratories for testing^3–5^. The ability to perform testing frequently and in the field with results available rapidly but with a low limit of detection is important because it permits self-isolation and quarantine early in the course of infection and can serve a “gating” function to limit entry of infected individuals into a high-risk environment, thereby preventing asymptomatic transmission^6–10^.

**Figure 1:**
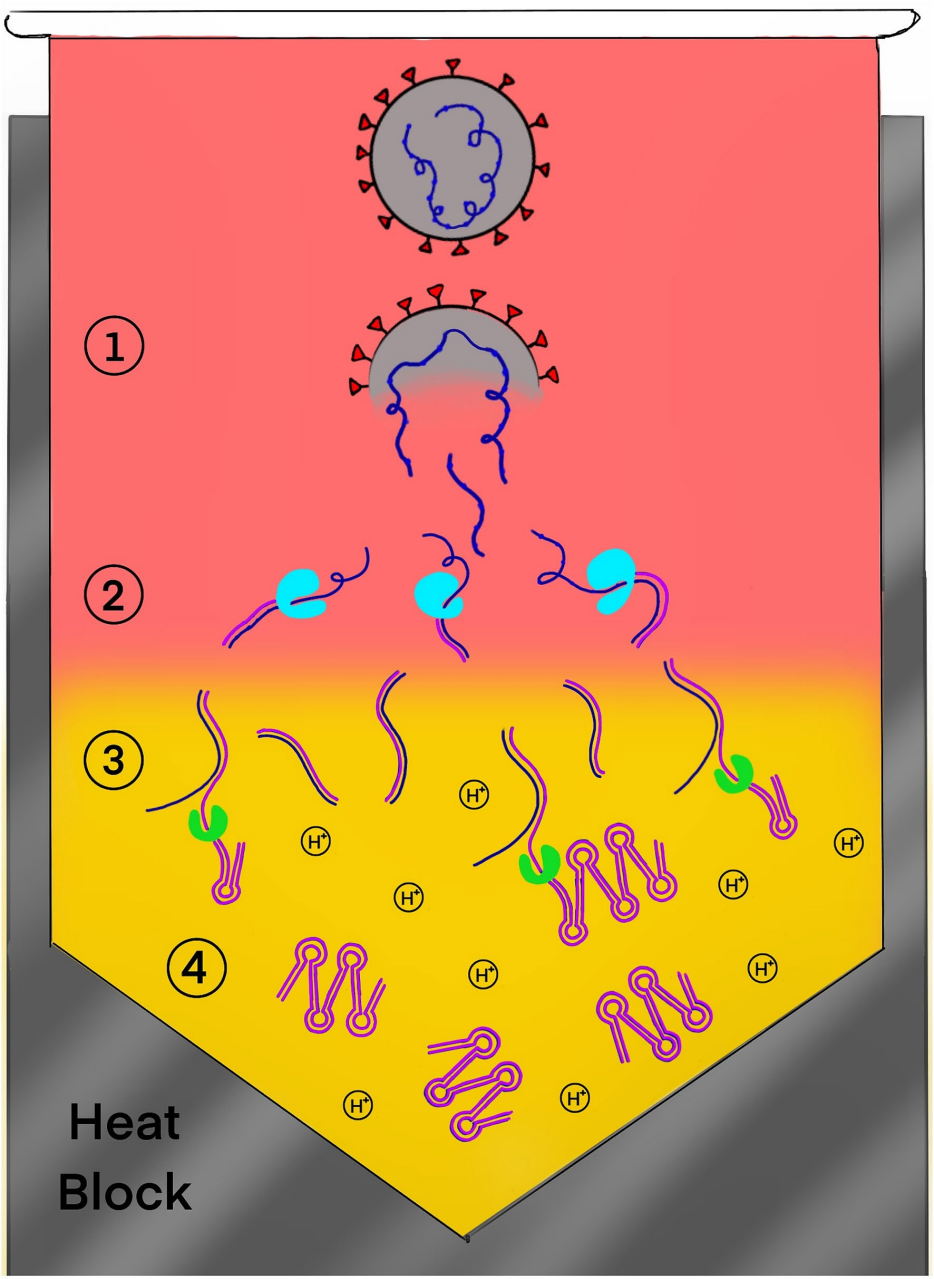
Diagram illustrating the principle of the HP-LAMP assay. Four steps occur within a single microcentrifuge tube containing the HP-LAMP cocktail. These steps consist of (1) Heat-mediated release of the single-stranded RNA virus (blue) from the virion, (2) Reverse transcription of the single-stranded viral RNA to generate cDNA (purple), (3) Loop-mediated amplification (LAMP) resulting in a decrease in pH, (4) Color change caused by decreased pH. The addition of RNA, DNA and RNAse inhibitors to the HP-LAMP cocktail inhibits degradation of the viral RNA by RNAses naturally found in saliva.

**Figure 2:**
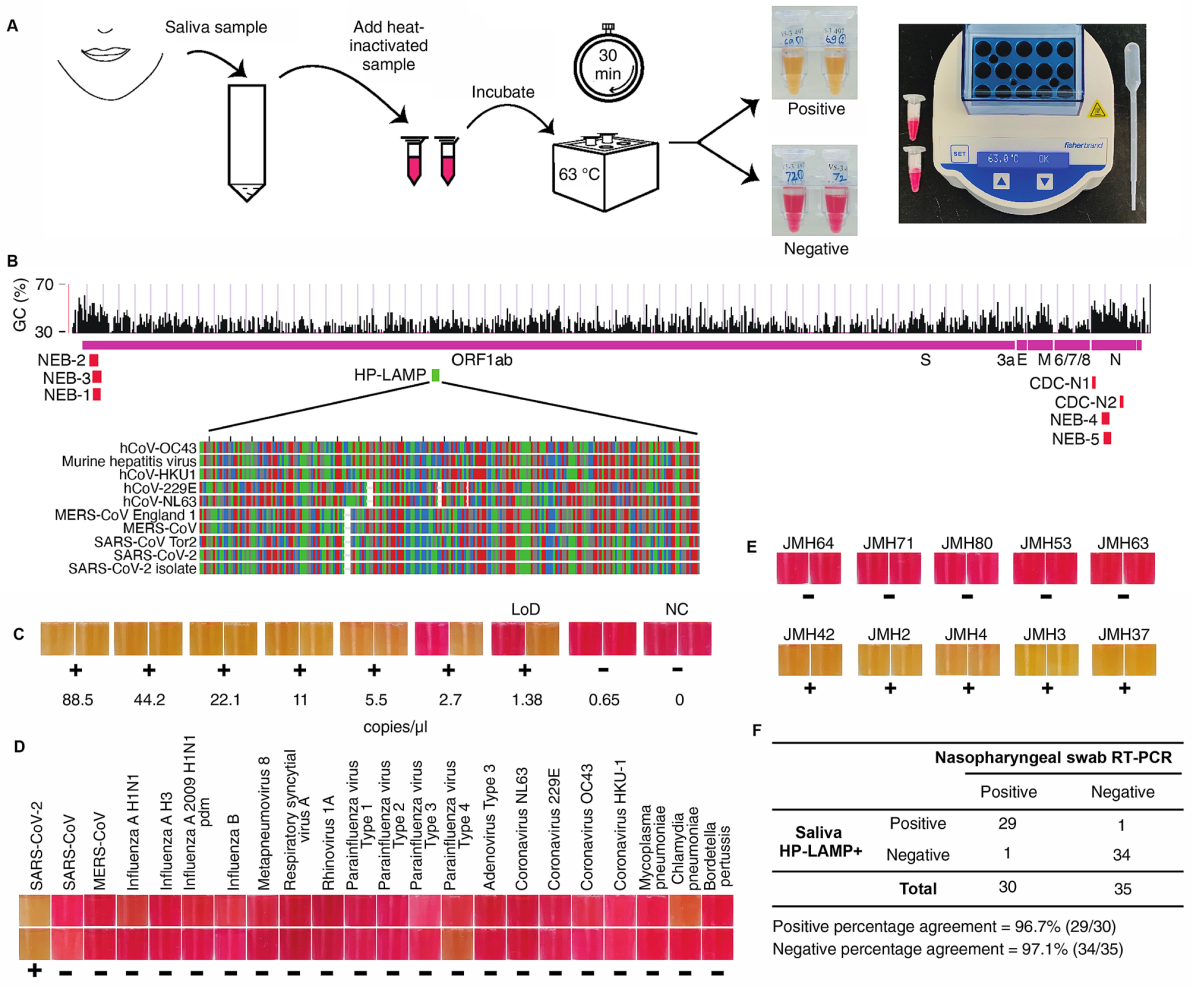
(**A**) HP-LAMP assay workflow. Heat-inactivated saliva (5 µl) was added to each of the two 1.5 ml microcentrifuge tubes pre-filled with the reaction mixture, incubated at 63°C for 30 min and then visualized for colorimetric change (yellow = positive; red = negative). At least one out of two tubes must turn yellow to interpret the assay result as positive. The minimum equipment needed to run the assay is a disposable transfer pipette, 2 heat blocks, and microcentrifuge tubes prefilled with reaction mixture. No prior RNA extraction or treatment is required. (**B**) Genome map showing targeted region of primers used for HP-LAMP in green. Locations of primers and probes from the CDC 2019-nCoV Real-Time RT-PCR Diagnostic Panel/New York SARS-CoV-2 Real-time RT-PCR Diagnostic Panel, and the New England Biolabs’ (NEB) SARS-CoV-2 assay are indicated in Red. Whereas previously used primers and probes targeted the GC-rich regions located at the 5′ and 3′ ends of the viral RNA, the primers used for HP-LAMP target the central portion of the viral RNA, which is better protected from digestion by salivary exonucleases. The targeted region of the HP-LAMP primer set with alignments of other Betacoronavirus genomes are featured^29,15^. Each nucleotide is shown (A: green; G: gray; T: red; C: blue). The percentage of GC-content across the genome is indicated (http://genome.ucsc.edu)^30,16^. (**C**) Determining the limit of detection (LoD) of the HP-LAMP assay. The concentrations indicated show copies of heat-inactivated SARS-CoV-2 per µl of saliva. NC = negative control with no SARS-CoV-2 added. ‘ + ’ = Positive HP-LAMP result; ‘-’ = Negative HP-LAMP result. The color of each box is taken directly from its corresponding reaction tube. (**D**) Cross-reactivity of HP-LAMP assay on common pathogens. Inactivated known respiratory pathogens (n = 21) along with inactivated SARS-CoV-2 virus were tested using HP-LAMP assay. All pathogens showed negative detection results in HP-LAMP assay, expect for SARS-CoV-2 virus. (**E**) Representative results of HP-LAMP testing on clinical samples. (**F**) Table shows positive and negative percentage agreement of HP-LAMP for detection of SARS-CoV-2 in saliva compared with nasopharyngeal swab RT-PCR results.

Reverse Transcription Loop-mediated isothermal Amplification (RT-LAMP), is a targeted isothermal nucleic acid amplification method that utilizes a combination of 2–3 primer sets and a DNA polymerase with high strand displacement activity^11^. While RT-LAMP has been used for SARS-CoV-2 detection by several groups^6,12–14^, these methods require a prior extraction step or lengthy sample treatment (which makes it difficult to deploy in the field), multiple fluid transfer steps, or lack the accuracy and limit of detection necessary for clinical implementation, and are therefore not suitable for clinical testing outside of a laboratory. Here we report the development and initial validation of a SARS-CoV-2 detection assay based on RT-LAMP, but with significant modifications made to enable detection of single-copy levels of virus in < 30 min directly from heat-inactivated saliva using only a single fluid transfer step and simple heat block with a simple colorimetric readout that can be interpreted with the unaided eye. We term the new assay High-Performance LAMP (HP-LAMP). A diagram illustrating the principle of HP-LAMP is shown in Fig. 1 and the workflow is illustrated in Fig. 2A.

To develop HP-LAMP, we first designed novel primers for targeting the SARS-CoV-2 virus. Existing primers used for RT-PCR and LAMP based nucleic acid testing of SARS-CoV-2 target the GC-rich regions located at the 5′ and 3′ ends of the virus. However, because salivary exonucleases degrade viral RNA from the ends, we designed our primers to target the central portion of the virus that would be better protected. We designed eight sets of six LAMP primers targeting SARS-CoV-2 reference genome (NC_045512.2) (Fig. 2B, Figure S1A). The central region of SARS-CoV-2 genome is GC-poor (AT-rich), making it difficult to select primer candidates across the genome with optimal annealing temperatures when following standard parameters for primer design. Therefore, we designed the primers to permit large primer-mediated loop-structures while ensuring that the primers did not form stable secondary structures or self-dimerize. We tested these primer sets along with previously published primer sets (Table S1)^12,17^, using serial dilutions of 500 to 0.5 copies of SARS-CoV-2 RNA standard spiked into a 25 μl standard RT-LAMP reaction (Figure S1A). The in-house designed primer set V5 detected 10^0^ to 10^−1^ copies level viral RNA in water, representing a 10- to 100-fold improvement in sensitivity and equivalent specificity compared with previously published primer sets (Figure S1A). *In-silico* inclusivity analysis of primer set V5 performed by aligning all primer sequences against all (n = 16,453) complete SARS-CoV-2 genomic sequences deposited in the NCBI Virus database on September 15, 2020 showed a 100% match for the ORF1ab gene was found for 98.8% of SARS-CoV-2 strains (n = 16,264), 1 mismatch was found to 1.2% (n = 182), and 2 mismatches were found to 0.04% (n = 7) of strains deposited in the NCBI Virus database^15^, respectively (Table S2). No instances of more than two mismatches were found. In silico cross-reactivity/exclusivity was performed by aligning the V5 primer sequences against sequences of 32 common viruses as well as coronaviruses related to SARS-CoV-2. Both the Forward Inner Primer (FIP) and the Backward Inner Primer (BIP) consist of 2 sections of non-continuous genomic sequences and were aligned separately to increase the sensitivity of alignment of cross-reactivity. In total, 6 primers corresponding to 8 sections of virus genome were assessed in silico for potential cross-reactivity against 32 common respiratory pathogens including six other human coronaviruses (SARS-CoV, MERS-CoV, HCoV-HKU-1, HCoV-NL63, HCoV-OC43 and HCoV-229E) (Table S3)^17^. None of the pathogens tested have a match against the total sequence length of the SARS-CoV-2 primers greater than the recommended threshold of 80%, except for SARS-CoV virus. The greatest percentage match is 92.0% on part 1 (~ 50%) of the FIP primer, and 95.8% on the Loop Backward (LB) primer against SARS-CoV virus^17^.

Next, we systematically modified the RT-LAMP reaction conditions to improve performance. We found that sensitivity and specificity of the assay could be markedly improved by adding carrier DNA, carrier RNA, and RNase inhibitors, as well as by increasing the reaction volume and introducing a heat-inactivation step (Figure S1B-J). Because of the risk that carry-over product from prior samples could cross contaminate a new sample and lead to false-positive results, we added Deoxyuridine Triphosphate (dUTP) and Antarctic Thermolabile uracil-DNA N-glycosylase (UDG) to our reaction mixture to incorporate dUTP into the HP-LAMP product and digest the HP-LAMP carry-over^17^.

To determine the limit of detection (LoD) of HP-LAMP, twofold serial dilutions of intact virus were spiked into negative saliva in concentrations ranging from 88.5 to 0.69 copies/µl of saliva (Fig. 2C). At the LoD of 1.38 copies/µl of saliva, 19/20 replicates (95%) were positively detected. At 2 × LoD (2.7 copies/ µl of saliva), 20/20 replicates (100%) were detected (Figure S2). This LoD is comparable to other U.S. Food and Drug Administration (FDA) Emergency Use Authorization (EUA) authorized swab- and saliva-based tests that must be run in centralized high complexity laboratories, including swab-based assays, such as LabCorp’s COVID-19 RT-PCR test (~ 15.625 copies/reaction), the Centers for Disease Control and Prevention (CDC) 2019-nCoV Real-Time RT-PCR panel (~ 10^0^ to −0.5 copies/µL)^18,19^, SalivaDirect (6 copies/µL), Fluidigm Corporation’s Advanta Dx (6.25 copies/µL), as well as rapid point-of-care swab tests, such Quidel Lyra Direct (34 copies/µL), though these were tested using different reference panels and thus direct comparison is difficult^18–25^. Wet testing for cross-reactivity/exclusivity was performed to evaluate potential cross-reactivity/exclusivity of the assay with 21 respiratory pathogens (Fig. 2D, Figure S3). All results, except for the SARS-CoV-2, of wet bench testing were negative (Fig. 2D, Figure S3).

Clinical evaluation of HP-LAMP was performed by comparing results from 65 blinded, paired, nasopharyngeal (NP) swab and saliva samples collected at the same time from symptomatic patients at Jackson Memorial Hospital (JMH) and Columbia University Irving Medical Center (CUIMC). Samples were collected throughout the day without the need for study subjects to be fasting or have previously rinsed their mouths. Samples containing food debris, thick mucus or frank blood were included in the analysis and were not excluded. A representative image of test results of some of the samples collected is shown in Fig. 2E. The testing showed that HP-LAMP had a positive percentage agreement (PPA) of 96.7% (95% CI = 82.8–99.9%) and negative percentage agreement (NPA) of 97.1% (95% CI = 85.1–99.9%) (Fig. 2F, Figure S4). The RT-PCR cycle threshold (Ct) values for SARS-CoV-2 target N2 from the NP swab from these positive samples ranged from to 14.2–41.6 (Table S5).

**Figure 3:**
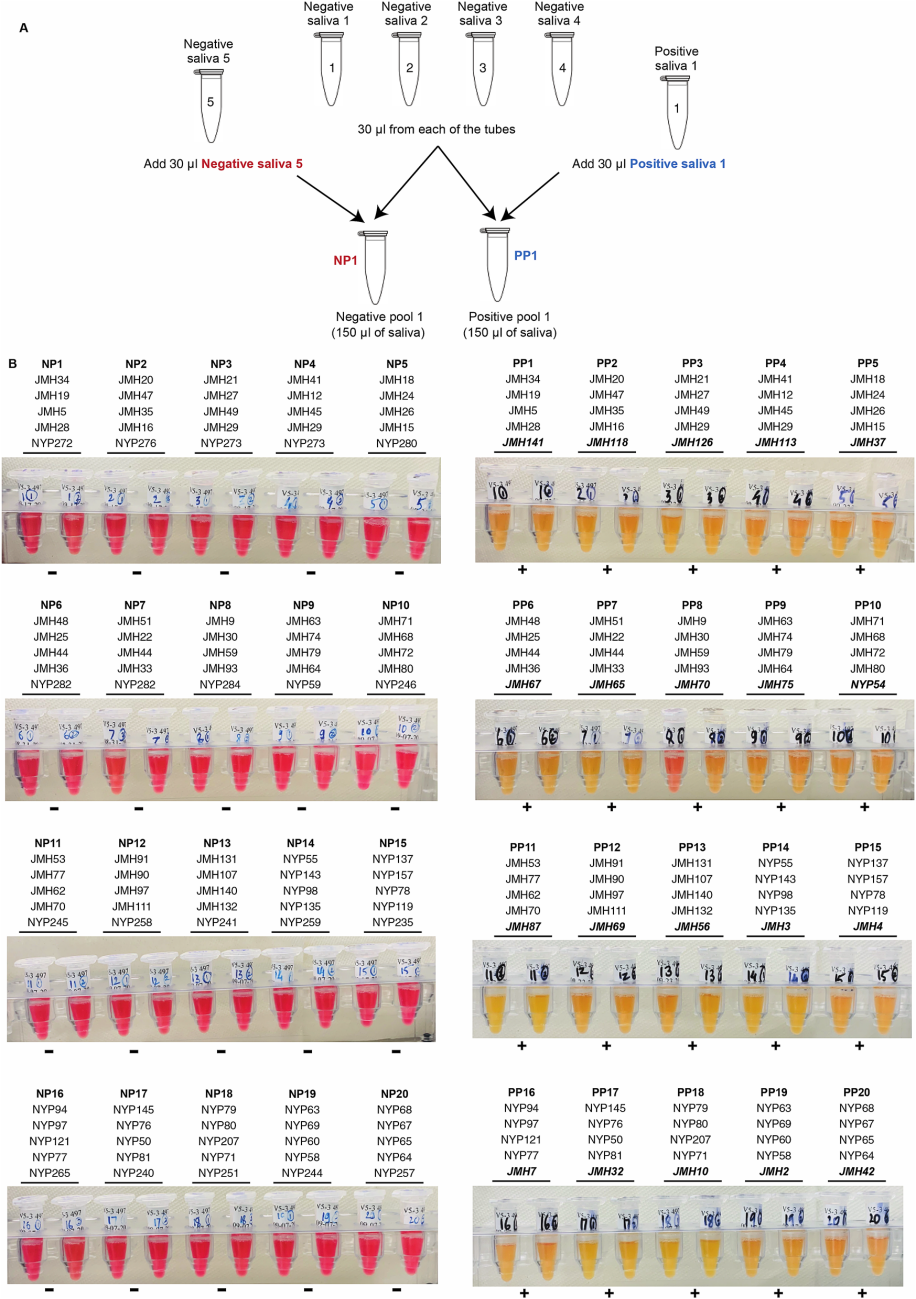
HP-LAMP assay for pooled samples. (**A**) Illustration of the HP-LAMP assay for sample pooling. A negative matrix was created by pooling 4 known negative saliva samples. A positive or negative pool of 5 samples (N = 5) was created by pooling a known positive or negative sample with the negative matrix. (**B**) HP-LAMP testing for pooled saliva samples. 20 positive (n = 20) and 20 negative (n = 20) pools were subjected to HP-LAMP assay.

Sample pooling allows multiple people to be tested at once in a single assay. This enables testing of more individuals in a shorter time using fewer resources and is, therefore, an important public health tool^26^. To evaluate the ability of HP-LAMP to be used with pooled samples, we tested pooling of five individual samples. The negative sample matrix was created by individually pooling 80 negative clinical samples into 20 pools of N = 4 before adding either a single positive or negative sample to create the final testing pool. Twenty positive pools and 20 negative pools of five were tested by HP-LAMP. HP-LAMP accurately detected 20/20 (100%) positive pools and 20/20 (100%) negative pools (Fig. 3).

The equipment costs for performing the HP-LAMP assay are very low; performing the assay requires only a pipette, a mini centrifuge, a vortexer, and two heat blocks that retail for ~ 250 USD each. In contrast, the equipment costs for RT-PCR based methods is > 45,000 USD while the automated Roche cobas 6800 unit costs ~ 350,000 USD^27,28^. If purchasing the consumable reagents individually using off-the-shelf components, the costs for the HP-LAMP assay is ~ 80 USD per assay, and ~ 16 USD per assay when pooling five samples. The cost for pre-made HP-LAMP cocktails is 20–25 USD per assay (Sorrento Therapeutics). The consumable cost for RT-PCR based methods is 20–60 USD per assay^27^.

In summary, HP-LAMP enables rapid detection of SARS-CoV-2 directly from saliva without the need for a lab, using a simple, one-step protocol. HP-LAMP has an LoD of < 2 viral copies per μl of saliva and a positive and negative percentage agreement of > 96% and > 97%, respectively, compared with RT-PCR testing of paired NP swab samples, comparable to the gold-standard RT-PCR based methods that must be run in a high-complexity laboratory. The simple workflow may also allow adaptation for at-home testing and pooling strategies. An FDA emergency use authorization (EUA) application is currently under review.

## Methods and materials

### Ethics

The study was reviewed and approved by the Columbia University Institutional Review Board (IRB) (#AAAS9893) and all methods were carried out in accordance with relevant guidelines and regulations. All study subjects signed informed consent prior to participating.

### Participant enrollment

Study participants were enrolled at New York Presbyterian Hospital when they underwent routine clinical testing for SARS-CoV-2 from 04/29/2020 to 06/1/2020 at the cough and fever clinic or a COVID-19 testing tent. Study participants were enrolled at Jackson Memorial Hospital (JMH) when they underwent routine clinical testing for SARS-CoV-2 upon presentation to the emergency room from 08/14/2020 to 09/10/2020.

### Sample collection

Nasopharyngeal (NP) swab and saliva samples were obtained from participants following CDC-recommended protocols. Nasopharyngeal swab samples were transported in 3 mL viral transport medium (VTM) and subjected to routine clinical testing for RT-PCR-based SARS-CoV-2 testing. Saliva samples were self-collected by each participant, by spitting ~ 1 mL of saliva into a clean 50 mL DNA LoBind Conical Tube (Eppendorf, 0,030,122,232). Saliva samples were shipped with −80°C ice packs and stored at −80°C until use. SARS-CoV-2 positive (n = 30) and SARS-CoV-2 negative (n = 35) samples were included in this study.

### Contrived samples for direct saliva testing

Contrived samples were prepared using SARS-CoV-2 Standard (200,000 cp/mL) (Exact Dx, COV019) spike-in or inactivated virus (ATCC, VR-1986HK). SARS-CoV-2 RNA Standard was diluted in nuclease-free water (Ambion, AM9937), and 1 to 100 copies of viral RNA were spiked into each reaction along with 5-20µL of saliva from healthy individuals as detailed below. Inactivated virus was spiked into saliva from healthcare workers who tested negative for SARS-CoV-2, and serially diluted to the targeted concentration using additional negative saliva.

### Primer design and screening

Primer sets, buffers, and incubation methods were systematically tested to develop the optimized method used herein that would be sufficiently sensitive and robust to enable direct detection of 10^0^ copies/µL viral RNA from saliva samples (Figure S1). PrimerExplorer V5 (https://primerexplorer.jp/e/) and the SARS-Cov-2 reference genome NC_045512v2 were used to design the HP-LAMP primers. The primers were matched against human reference genome Hg19 and Human Coronavirus reference genome to ensure specificity^29,30^ (Fig. 2B, Figure S1A). Typically, primers for LAMP are designed to target GC-rich regions of the viral RNA because GC-rich regions bind more tightly to primers. However, in SARS-CoV-2, these regions are found towards the 5′ and 3′ ends of the viral RNA. To target the GC-poor (AT-rich) regions in the center portion of SARS-CoV-2 genome, we designed the primers to permit large primer-mediated loop-structures while ensuring that the primers did not form stable secondary structures or self-dimerize. We also aligned the known SARS-CoV-2 genomic sequence with those of six other human coronaviruses (SARS-CoV, MERS-CoV, HCoV-HKU-1, HCoV-NL63, HCoV-OC43 and HCoV-229E) to ensure no cross-reactivity.

In-house and published primers^12,17^ were screened using a standard RT-LAMP Protocol (NEB, M1800) (Figure S1A) with the addition of 0.14 µM dUTP and 0.0002 unit/µL Antarctic Thermolabile UDG (NEB, M0372S). Primer set V5 was designed to target a central portion of the SARS-CoV-2 genome and was able to detect 10^0^ copies of viral RNA per reaction with no false positive amplification in the negative control (Figure S1A). It was used for further development of HP-LAMP assay, and is termed as HP-LAMP primer set (Fig. 2B).

The sequences for the primers used are shown in Table S1. CDC 2019-Novel Coronavirus (2019-nCoV) Real-time RT-PCR Primers were also included as a reference^31^.

### Preparing one-step RT-LAMP reaction master mix (HP-LAMP)

A 25-fold primer mix of LAMP primers (V5.FIP, V5.BIP, V5.LF, V5.LB, V5.F3, V5.B3; Table S1) was prepared by assembling 40 µM FIP and BIP, 10 µM V5.LF and V5.LB, and 5 µM V5.F3 and V5.B3 primers in nuclease-free water (Ambion, AM9937). A 2 × colorimetric RT-LAMP master mix was prepared by adding 3.5µL 100 mM dUTP (Thermo Scientific, R0133), 0.5µL Antarctic Thermolabile UDG (NEB, M0372S), and 0.25µL 5 mM SYTO 9 (Invitrogen, S34854) to 1,250µL WarmStart Colorimetric LAMP 2 × Master Mix (DNA & RNA) (NEB, M1800S/L). The final reaction mix for one reaction includes 250µL 2 × colorimetric RT-LAMP master mix, 20µL 25-fold LAMP primer mix, and 190µL nuclease-free water, 20 µL of lysis buffer ((0.1% tween-20, 2% volume (i.e., 2µL added to 100µL) ezDNase (Invitrogen, 11,766,051)), 0.3 ng/µL lysis buffer volume of carrier DNA (human genomic DNA from a normal male e.g., 6 ng carrier DNA for 20µL lysis buffer), and ~ 9 ng/µL lysis buffer volume of carrier RNA (NEB, N0362S, ~ 250 ng/µL), 2µL RNase Inhibitor, Murine (NEB, M0314S/L), 15µL buffer TE pH 8.0 (Ambion AM9849), and can be scaled up according to the actual number of samples. Lysis buffer was mixed with the carrier gDNA and incubated at RT for ~ 15 min before use. For each reaction, 497µL of the final reaction mix was preloaded in a clean 1.5 mL LoBind microcentrifuge tube (Eppendorf, 022,431,021), stored at −20°C, and thawed at 4°C before use. This is the final reaction mix used for the HP-LAMP assay, and each sample was tested in duplicate. HP-LAMP assay was QC'ed using negative saliva with 1–2 × LoD inactivated SARS-CoV-2 virus spike-in, or 25 copies SARS-CoV-2 virus RNA standard.

### SARS-CoV-2 detection on saliva samples using HP-LAMP without RNA extraction

Saliva samples were subjected to a 95°C heat inactivation for 5 min^32,33^, and then cooled on ice. 5µL of saliva sample was added to the one-step HP-LAMP final reaction mix, mixed by gentle pipetting using a transfer pipette (Fisherbrand, 13-711-20), and incubated at 63°C for 30 min in a portable heat block (Fisherbrand, 14-955-219). The reaction was paused by placing on ice for 1 min, and the colorimetric results were then recorded visually and by camera (Figure S5).

### Determining the limit of detection (LoD)-analytical sensitivity

The limit of detection (LoD) is defined as the lowest concentration at which 19/20 replicates (or approximately 95% of all true positive replicates) are positively detected. To determine the LoD of HP-LAMP, intact SARS-CoV-2 (ATCC VR-1986HK, Batch 70,037,676) with a known virus concentration (1.77 × 10^5^ copies/μl) was spiked into saliva from healthcare workers who tested negative for SARS-CoV-2 using the Roche cobas system. The following twofold dilution series was tested: 88.5, 44.2, 22, 11, 5.5, 2.75, 1.38, and 0.69 copies/μl of saliva. The dilutions of 5.5, 2.75, 1.38, and 0.69 copies/μl were tested in triplicate to determine the ‘preliminary LoD’. Spiked saliva specimens were tested according to protocol for the HP-LAMP Assay. The preliminary LoD was then confirmed with 20 additional replicates (Figure S2). The LoD of the HP-LAMP Assay was determined to be 1.38 copies/μl of saliva. At this LoD, 19/20 (95%) individual replicates at a concentration of 1.38 copies/μl of saliva tested positive (Figure S2).

### Determining inclusivity (analytical sensitivity)

An in silico inclusivity analysis was performed by aligning all primer sequences against all (n = 16,453) complete SARS-CoV-2 genomic sequences deposited in the NCBI Virus database on Sep 15, 2020^15^. The HP-LAMP primer set 100% matched to the ORF1ab gene in 98.8% of SARS-CoV-2 strains and had 1 mismatch to 1.2%, and 2 mismatches to 0.04% of strains deposited in the NCBI Virus database, respectively (Table S2).

### Determining cross-reactivity (analytical specificity)

In silico cross-reactivity was performed by aligning the HP-LAMP primer sequences against sequences of common viruses as well as coronaviruses related to SARS-CoV-2 using NCBI Blast. Both primer FIP and BIP consist of 2 sections of non-continuous genomic sequences and were aligned separately to increase the sensitivity of alignment of cross-reactivity. In total, 6 primers corresponding to 8 sections of virus genome were searched against 32 common pathogens (Table S3)^17^. No pathogen except for SARS-CoV shared ≥ 80% with the primer sequences. SARS-CoV shared 92.00% on part 1 (~ 50%) of FIP primer, and 95.80% on LB primer.

Wet testing was performed to evaluate potential cross-reactivity/exclusivity of the assay with other organisms using ZeptoMetrix Corporation NATtrol Respiratory Verification Panel (ZeptoMetrix, NATRVP-IDI) including 19 respiratory pathogens, NATtrol Coronavirus-SARS Stock (ZeptoMetrix, NATSARS-ST), NATtrol MERS-CoV Stock (ZeptoMetrix, NATMERS-ST), and NATtrol SARS-Related Coronavirus 2 (SARS-CoV-2) External Run Control (ZeptoMetrix, NATSARS(COV2)-ERC). Samples were prepared by spiking 3µL inactivated, intact viral particles or bacterial cells using the panels/organisms into negative saliva samples and were subsequently processed using HP-LAMP. Virus and bacteria were tested at concentrations similar to or greater than the SARS-CoV-2 virus External Run Control (50,000 copies/mL). All the results of wet bench testing, except for that of SARS-CoV-2, were negative (Table S4, Figure S3).

### Clinical evaluation

The performance of HP-LAMP was compared to test results from paired nasopharyngeal (NP) swab samples. The study was conducted with symptomatic patients from Jackson Memorial Hospital (JMH) and Columbia University Irving Medical Center (CUIMC) who each provided a paired NP and saliva sample on the same day. NP samples were immediately processed in the clinical pathology laboratory using FDA authorized Roche cobas^34^, Cepheid^35^, Qiagen^36^, or EliTech (GendFinder)^37^ systems for SARS-CoV-2 testing at JMH and CUIMC (depending on the available testing option at the time of testing). Saliva was collected in blinded sterile tubes (Eppendorf, 0,030,122,232) without any preservatives and sent to Columbia University Fertility Center for testing by HP-LAMP. A total of 65 samples were tested: 30 samples that were positive for SARS-CoV-2 by NP swab and 35 that were negative by NP swab. After testing, results were sent back to JMH for unblinding (Figure S4). Samples containing food particles or blood were not excluded.

### Sample pooling

Sample pooling of 5 samples (N = 5) was performed by combining 30µL from 4 known negative saliva samples with either 30µL of saliva from a positive sample to create a positive pool of 5 samples or 30µL of saliva from a known negative sample to create a negative pool of 5 samples. 20 known positive samples (N2 Ct < 33) and 100 known negative samples were used to generate 20 positive pools and 20 negative pools for evaluation of pool testing using HP-LAMP assay.

## Supplementary Information

Supplementary Information
