# Supplementary material for: Field-deployable, rapid diagnostic testing of saliva for SARS-CoV-2
Source: Sci Rep. 2021 Mar 9;11:5448. doi: 10.1038/s41598-021-84792-8 (PMC7943555; doi:10.1038/s41598-021-84792-8)
Supplement: Supplementary file 1 — Supplementary Information [file 41598_2021_84792_MOESM1_ESM.pdf]

Supplemental materials

Field-deployable, rapid diagnostic testing of saliva for SARS-CoV-2

Shan Wei[1]*, Hemant Suryawanshi[1]*, Alexandre Djandji[1], Esther Kohl[1], Stephanie Morgan[1], Eldad A. Hod[1,2], Susan Whittier[1,2], Kevin Roth[1,2], Raymond Yeh[1], Juan Carlos Alejaldre[1,2], Elaine Fleck[1,2], Stephen Ferrara[1,2], Daniel Hercz[3], David Andrews[3], Lilly Lee[3], Kristopher A. Hendershot[3], Joshua Goldstein[3], Yousin Suh[1], Mahesh Mansukhani[1,2], and Zev Williams[1,2]**

[1] Columbia University Irving Medical Center, New York, NY, USA

[2] New York Presbyterian Hospital, New York, NY, USA

[3] Jackson Memorial Hospital, Miami, FL, USA

* These authors contributed equally

** Corresponding author

Email: zw2421@cumc.columbia.edu

Table S1. Sequence information of primers tested in this study

Primer set	ID	Primer	Reference
**V1**	V1.F3	CTGTCTATCCAGTTGCGTCA	This study
	V1.B3	GAATTGTGACATGCTGGACAA	
	V1.FIP	CTGCCATGAAGTTTCACCACAATGA-CCAAATGAATGCAACCAAATGTG	
	V1.BIP	CCACTTGCGAATTTTGTGGCAC-ACAACAGCATTTTGGGGTAAG	
	V1.LF	TCACACTTCATGAGAGTTGAAAGG	
	V1.LB	TGAGAATTTGACTAAAGAAGGTGCC	
**V2**	V2.F3	CCAGCATGTCACAATTCAGAAG	This study
	V2.B3	GTATGGTTACAACCTATGTTAGCG	
	V2.FIP	CCACCCTTACGAAGAATGGTTTTCA-TAGGACCTGAGCATAGTCTTG	
	V2.BIP	CGCACTATTGCCTTTGGAGGC-CTAGCACGTGGAACCCAAT	
	V2.LF	AGCCAGATTCATTATGGTATTCGG	
	V2.LB	ATGTTGGTTGCCATAACAAGTGTG	
**V3**	V3.F3	TGAGGATGAAGAAGAAGGTGATTG	This study
	V3.B3	AGTTGTCTGATTGTCCTCACTG	
	V3.FIP	GGCACCAAATTCCAAAGGTTTACCT-TGAAGAAGAAGAGTTTGAGCCATC	
	V3.BIP	TCTGCTGCTCTTCAACCTGAAGAA-CCGTCTTGTTGACCAACAGT	
	V3.LF	GGTAATCATCTTCAGTACCATACTCAT	
	V3.LB	GCAAGAAGAAGATTGGTTAGATGATG	
**V4**	V4.F3	TGCACTTATCTTAGCCTACTGTA	This study
	V4.B3	CACGTACAAGGTATCTGAACAC	
	V4.FIP	CCACGTTCAAGACTCTTTTGCAAGA-GGTGAGTTAGGTGATGTTAGAGAA	
	V4.BIP	TGTGTAAAACTTGTGGACAACAGCA-TTCATAAGAAAGTGTGCCCATG	
	V4.LF	TGGCATGTTGAAACAAGTAACTCAT	
	V4.LB	CCCTTAAGGGTGTAGAAGCTGTT	
**V5 (HP-LAMP)**	V5.F3	TGGATACAACTAGCTACAGAGAAG	15
	V5.B3	AGCCAAAGACCGTTAAGTGTA	
	V5.FIP	GTGGTGGTTGGTAAAGAACATCAGA-CTTGTTGTCATCTCGCAAAGG	
	V5.BIP	CCTCTATCACCTCAGCTGTTTTGC-TGTACCATACAACCCTCAACTT	
	V5.LF	ACCTGAGTTACTGAAGTCATTGAGA	

	V5.LB	TGGTTTTAGAAAAATGGCATTCCC	
**V6**	V6.F3	GGATGTTAACTGCACAGAAGTC	This study
	V6.B3	GCCGAGGAGAATTAGTCTGAG	
	V6.FIP	GCCTGCACGTGTTTGAAAAACATTA-CCTGTTGCTATTCATGCAGATC	
	V6.BIP	AATAGGGGCTGAACATGTCAACAAC-TCTGATAACTAGCGCATATACCTG	
	V6.LF	GAACCTGTAGAATAAACACGCCAAG	
	V6.LB	CATATGAGTGTGACATACCCATTGG	
**V7**	V7.F3	CCCCGCATTACGTTTGGT	This study
	V7.B3	TTGGAACGCCTTGTCCTC	
	V7.FIP	TTGTTTTGATCGCGCCCCAC-CCCTCAGATTCAACTGGCA	
	V7.BIP	CGGCCCCAAGGTTTACCCAA-GGAATTTAAGGTCTTCCTTGCC	
	V7.LF	TGCGTTCTCCATTCTGGTTAC	
	V7.LB-Lo	CTTGGTTCACCGCTCTCACTC	
**V8**	V8.F3	TCACGTAGTCGCAACAGTT	This study
	V8.B3	GAAGCCTCAGCAGCAGAT	
	V8.FIP	CAAAGCAAGAGCAGCATCACCG-GCAGCAGTAGGGGAACTT	
	V8.BIP	CTGCTTGACAGATTGAACCAGCT-CTTAGTGACAGTTTGGCCTTG	
	V8.LF	TGCCAGCCATTCTAGCAGGAG	
	V8.LB	TGAGAGCAAAATGTCTGGTAAAGG	
**1a-A (NEB-1)**	ORF1a-A-F3	CTGCACCTCATGGTCATGTT	12
	ORF1a-A-B3	AGCTCGTCGCCTAAGTCAA	
	ORF1a-A-FIP	GAGGGACAAGGACACCAAGTGTATGGTTGAGCTGGTAGCAGA	
	ORF1a-A-BIP	CCAGTGGCTTACCGCAAGGTTTTAGATCGGCGCCGTAAC	
	ORF1a-A-LF	CCGTACTGAATGCCTTCGAGT	
	ORF1a-A-LB	TTCGTAAGAACGGTAATAAAGGAGC	
**1a-B (NEB-2)**	ORF1a-B-F3	TCATCAAACGTTCGGATGCT	12
	ORF1a-B-B3	TATGGCCACCAGCTCCTT	
	ORF1a-B-FIP	CGACCGTACTGAATGCCTTCGAGAACTGCACCTCATGGTCAT	
	ORF1a-B-BIP	AGACACTTGGTGTCCTTGTCCCAGAAGAACCTTGCGGTAAGC	
	ORF1a-B-LF	CTGCTACCAGCTCAACCATAAC	
	ORF1a-B-LB	TCATGTGGGCGAAATACCAGT	
**1a-C**	ORF1a-C-F3	CTGCACCTCATGGTCATGTT	12

**(NEB-3)**	ORF1a-C-B3	GATCAGTGCCAAGCTCGTC	
	ORF1a-C-FIP	GAGGGACAAGGACACCAAGTGTGGTAGCAGAACTCGAAGGC	
	ORF1a-C-BIP	CCAGTGGCTTACCGCAAGGTTTTAGATCGGCGCCGTAAC	
	ORF1a-C-LF	ACCACTACGACCGTACTGAAT	
	ORF1a-C-LB	TTCGTAAGAACGGTAATAAAGGAGC	
**N-A (NEB-4)**	GeneN-A-F3	TGGCTACTACCGAAGAGCT	12
	GeneN-A-B3	TGCAGCATTGTTAGCAGGAT	
	GeneN-A-FIP	TCTGGCCCAGTTCCTAGGTAGTCCAGACGAATTCGTGGTGG	
	GeneN-A-BIP	AGACGGCATCATATGGGTTGCACGGGTGCCAATGTGATCT	
	GeneN-A-LF	GGACTGAGATCTTTCATTTTACCGT	
	GeneN-A-LB	ACTGAGGGAGCCTTGAATACA	
**N-B (NEB-5)**	GeneN-B-F3	ACCGAAGAGCTACCAGACG	12
	GeneN-B-B3	TGCAGCATTGTTAGCAGGAT	
	GeneN-B-FIP	TCTGGCCCAGTTCCTAGGTAGTTCGTGGTGGTGACGGTAA	
	GeneN-B-BIP	AGACGGCATCATATGGGTTGCACGGGTGCCAATGTGATCT	
	GeneN-B-LF	CCATCTTGGACTGAGATCTTTCATT	
	GeneN-B-LB	ACTGAGGGAGCCTTGAATACA	
**2019-nCoV_N1 (CDC-N1)**	2019-nCoV_N1-F	GACCCCAAAATCAGCGAAAT	27
	2019-nCoV_N1-R	TCTGGTTACTGCCAGTTGAATCTG	
**2019-nCoV_N2 (CDC-N2)**	2019-nCoV_N2-F	TTACAAACATTGGCCGCAAA	27
	2019-nCoV_N2-R	GCGCGACATTCCGAAGAA	

FIP: forward inner primer; BIP: backward inner primer; LF: loop forward; LB: loop backward.

Table S2. in silico Inclusivity analysis

Characteristics	CUFC-primers (Orf1ab gene)
**Total primer length (nt)**	186
**Total # of Strains Evaluated**	16453
**100% Match***	16264
**1 Mismatch***	182
**2 Mismatches***	7
**3 Mismatches**	0
**>3 Mismatches**	0

*Some isolate genome sequences contain degenerate bases in the region of primers. Currently, if the degenerate base match appears in the middle of a primer, and the degenerate base include the base present on the primer according to the IUPAC degenerate nucleotide codes (e.g., R=A or G, the isolate genome sequence has R and the primer has A), it's counted as match (18 isolates in the 100% match category). If the degenerate base match appears at the 3'end of a primer (which in general has greater impact on amplification efficiency), and the degenerate base includes the base present on the primer according to the IUPAC degenerate nucleotide codes, it's counted as a mismatch in this table. Degenerate bases match in the middle of 2 primers are counted as mismatch (2 isolates in the 2 mismatches category).

Table S3. Cross-reactivity in silico analysis

Pathogen	F3	B3	FIP part 1	FIP part 2	BIP part 1	BIP part 2	LF	LB
Human adenovirus C	no match	no match	no match	no match	no match	no match	no match	no match
Human metapneumovirus isolate 00-1	no match	no match	no match	no match	no match	no match	no match	no match
Human parainfluenza virus HPIV-1 strain Washington/1964	no match	no match	no match	no match	no match	no match	no match	no match
Human parainfluenza virus 4a	no match	no match	no match	no match	no match	no match	no match	no match
Human parainfluenza virus 4a strain HPIV4a/Seattle/USA/SC9971/2018	no match	no match	no match	no match	no match	no match	no match	no match
Human parainfluenza virus 4b strain HPIV4b/Seattle/USA/SC0496/2019	no match	no match	no match	no match	no match	no match	no match	no match
Human parainfluenza virus 2 strain G REER	no match	no match	no match	no match	no match	no match	no match	no match
Human parainfluenza virus 3	no match	no match	no match	no match	no match	no match	no match	no match
Human enterovirus 68 strain Fermon	no match	no match	no match	no match	no match	no match	no match	no match
Influenza A virus (A/California/07/2009(H1N1))	no match	no match	no match	no match	no match	no match	no match	no match
Influenza B virus (B/Lee/1940)	no match	no match	no match	no match	no match	no match	no match	no match
Respiratory syncytial virus	no match	no match	no match	no match	no match	no match	no match	no match
Human rhinovirus A1	no match	no match	no match	no match	no match	no match	no match	no match
Chlamydia pneumoniae TW-183 (chlamydias)	no match	no match	no match	no match	no match	no match	no match	no match
Haemophilus influenzae ASM76707v1	no match	no match	no match	no match	no match	no match	no match	no match
Legionella pneumophila ASM194158v1	no match	no match	no match	no match	no match	no match	no match	no match
Mycobacterium tuberculosis H37Rv (high GC Gram+)	no match	no match	no match	no match	no match	no match	no match	no match
Streptococcus pneumoniae R6	no match	no match	no match	no match	no match	no match	no match	no match
Streptococcus pyogenes M1 GAS	no match	no match	no match	no match	no match	no match	no match	no match
Bordetella pertussis 18323	no match	no match	no match	no match	no match	no match	no match	no match
Mycoplasma pneumoniae M129	no match	no match	no match	no match	no match	no match	no match	no match
Candida albicans SC5314	no match	no match	no match	no match	no match	no match	no match	66.70%
Pseudomonas aeruginosa PAO1	no match	no match	no match	no match	66.70%	no match	no match	no match
Pneumocystis jirovecii (ascomycetes)	no match	no match	no match	no match	no match	no match	no match	no match
Staphylococcus epidermidis ATCC 12228	no match	no match	no match	no match	no match	no match	no match	no match
Streptococcus salivarius ASM78551v1	no match	no match	no match	no match	no match	no match	no match	no match
Human coronavirus 229E	no match	no match	no match	no match	no match	no match	no match	no match
Human coronavirus OC43 strain ATCC VR-759	no match	no match	no match	no match	no match	no match	no match	no match
Human coronavirus HKU1	no match	no match	no match	no match	no match	no match	no match	no match
Human coronavirus NL63	no match	no match	no match	no match	no match	no match	no match	no match
MERS-coronavirus	no match	no match	83.30%	no match	no match	no match	no match	no match
SARS-coronavirus	no match	no match	92.00%	no match	no match	no match	no match	95.80%
SARS-CoV-2	100%	100%	100%	100%	100%	100%	100%	100%

Table S4. Cross-activity wet lab testing

Organism/Strain	ZMC Panel Specs (Ct)	HP-LAMP+ Testing result
Influenza A H3N2	25-28	Neg
Influenza A H1N1	22-25	Neg
Influenza A 2009 H1N1	22-25	Neg
Influenza B	21-24	Neg
Metapneumovirus 8	26-29	Neg
RSV A Stock	26-29	Neg
Parainfluenza 1	25-28	Neg
Parainfluenza 2	27-30	Neg
Parainfluenza 3	27-30	Neg
Parainfluenza 4	22-25	Neg
Rhinovirus 1A	25-28	Neg
Adenovirus 3	22-25	Neg
Coronavirus OC43	25-28	Neg
Coronavirus 229E	25-28	Neg
Coronavirus NL63	25-28	Neg
Coronavirus HKU-1	22-25	Neg
Bordetella pertussis	22-25	Neg
Chlamydophilia pneumoniae	25-28	Neg
Mycoplasma pneumoniae	22-25	Neg
MERS-CoV	25-28	Neg
SARS-CoV	25-28	Neg
Negative	N/A	Neg
SARS-CoV-2	27.5-29.5	Pos

ZMC: ZeptoMetrix Corporation.

Table S5. Target N2 Ct value of swab positive samples

Platform	Ct (target N2 or N)
CEPHEID	16.4
CEPHEID	19.2
CEPHEID	21.0
CEPHEID	23.9
CEPHEID	24.3
CEPHEID	26.7
CEPHEID	26.7
CEPHEID	30.5
CEPHEID	31.4
CEPHEID	34.3
CEPHEID	40.1
CEPHEID	41.6
CEPHEID	41.6
GeneFinder	16.33
GeneFinder	18.87
GeneFinder	24.31
GeneFinder	24.54
GeneFinder	28.38
GeneFinder	30.01
QIAGEN	14.2
QIAGEN	17.72
QIAGEN	24.64
QIAGEN	29.71
QIAGEN	30.89
QIAGEN	32.79
QIAGEN	33.16

Supplementary Figure Legends

Figure S1. Development of HP-LAMP.
(Colorimetric results: yellow=positive; red=negative; intermediate=negative)

A) Genome map showing targeted region of primers. Primers from previous publication are indicated in red (1a-A, 1a-B, 1a-C, N-A, N-B). In-house designed primer sets are indicated in green (V1-8). The targeted region of primer set V5 with alignments of other Betacoronavirus genomes are featured[27,36]. Each nucleic acid is shown (A: green; G: gray; T: red; C: blue). The percentage of GC-content across the genome is indicated[28,37]. The middle panel shows screening primer sets for SARS-CoV-2 testing. Those primer sets with sensitivities of 500 copies or fewer of SARS-CoV-2 are shown. A negative control and between 0.5 – 500 copies of SARS-CoV-2 RNA were spiked into 25 µL reaction volume and assayed using RT-LAMP with previously reported primer sets (1a-A, N-A, N-B) and in-house designed primer sets (V3, V5 (ultimately the HP-LAMP primer), V7, V8). Color blocks reflect the actual color captured from a 96-well PCR plate. The right panel shows comparison of standard RT-LAMP and HP-LAMP sensitivity. Contrived saliva samples containing 0.25-200 copies of SARS-CoV-2 viral RNA per µL of saliva, along with a negative control, were tested using the V5 primer set with RT-LAMP and HP-LAMP. While RT-LAMP failed to detect viral RNA at levels as high as 200 copies/ µL of saliva, HP-LAMP consistently detected levels as low as ~1 copy/µL of saliva, and often less. Color blocks represent the actual colorimetric results.

B) Carrier DNA increase RT-LAMP specificity in large reaction volume. Large reaction volume led to longer time to reach amplification plateau and higher chances of non-template amplification. V5 primer set rapidly detected the presence of 5 copies/µL SARS-CoV-2 RNA in 30min in large volume, and the presence of carrier DNA helped to maintain specificity in 45min incubation. N-A primer and a multiplex of N-A primer and V5 primer failed to detect viral target in 30min, and hence required extended incubation to 45min. At 45min, the negative control with 0.3ng/µL carrier DNA remained true negative.

C) Effects of RNase inhibitor on RT-LAMP. RNAase inhibitor (RI; 0 to 50% sample volume) was added to a RT-LAMP reaction. LAMP tolerated the presence of up to 25% volume of sample without a decrease in detection sensitivity and specificity. An addition of 10% RNase inhibitor showed comparable detection sensitivity and specificity to no RNase inhibitor added.

D) Effects of 10% RNase inhibitor supplement on RT-LAMP in large reaction volumes. LAMP can directly detect 1 to 0.125 SARS-CoV-2 RNA copies/µL saliva with the presence of 10% volume RI in assay (i.e., 2µL RNase inhibitor for 20µL saliva).

E) Effect of carrier RNA on RT-LAMP. 0.00009 to 9 ng/µL carrier RNA was added to each reaction. 9 ng/µL carrier RNA showed the highest detection sensitivity.

F) Effect of carrier RNA on RT-LAMP in large reaction volume. Supplement of ~9 ng/µL carrier RNA in HP-LAMP resulted in detection of 0.25 SARS-CoV-2 RNA copies/µL saliva directly.

G) HP-LAMP detection of SARS-CoV-2 RNA in viral transport medium (VTM). HP-LAMP detected 5 to 0.5 viral copies/µL VTM. Saliva is more inhibitory to RT-LAMP reaction than VTM, hence the HP-LAMP assay is efficient to alleviate inhibitory effects of VTM and detect SARS-CoV-2 directly.

H) Effect of buffer TE and reduced sample volume. Addition of 15µL buffer TE and reduction of saliva input from 20µL to 5µL reduced the chance of false positive on clinical samples.

I) Detection sensitivity is maintained with 5µL saliva input. The detection sensitivity using 5µL is within 10^0 copies/µL saliva. The positive detection by colorimetric results were present after 26min-30min incubation at 63˚C.

J) Detection sensitivity and specificity improved with 5min heat-inactivation. Clinical saliva samples vary widely and are impacted by many factors including the presence of food particles, overall health, body chemistry, blood and hydration. Some samples initially showed false negative results even with high viral load in saliva; some negative samples showed unexplained false positive or intermediate results. 95˚C 5min heat-inactivation eliminated the background effects and corrected 3 false negative or inconclusive clinical saliva samples into true positive and true negative. Heat inactivation also enhanced the biosafety of the test. It was incorporated in standard HP-LAMP assay for clinical validation.

Figure S2. Determining Limit of Detection (LoD) of HP-LAMP assay.

A) Scheme of performing 2-fold serial serial dilution.

B). Preliminary LoD. The preliminary range of LoD was determined by testing the following 2-fold dilution series: 88.5, 44.2, 22, 11, 5.5, 2.75, 1.38, and 0.69 copies/μl of saliva. The dilutions of 5.5, 2.75, 1.38, and 0.69 copies/μl were then tested in triplicate to determine 'preliminary LoD'.

C). Confirming the LoD. At 2.75 copies/µL, 20/20 samples were detected by HP-LAMP. At the LoD of 1.38 copies/µL, 19/20 samples (or approximately 95% of all true positive replicates) were positively detected.

Figure S3. Cross-reactivity of HP-LAMP assay on common pathogens.
Inactivated known respiratory pathogens (n=21) along with inactivated SARS-CoV-2 virus were tested using HP-LAMP assay. All pathogens showed negative detection results in HP-LAMP assay, expect for SARS-CoV-2 virus.

Figure S4. HP-LAMP testing results on clinical saliva samples.
Blinded clinical samples (n=65) were subjected to HP-LAMP assay, and the testing results were compared with paired RT-PCR results from nasopharyngeal swabs.

Figure S5. Interpretation of HP-LAMP colorimetric results. Yellow: positive; Red: negative; orange: negative. HP-LAMP is tested in duplicate, at least 1/2 tube turning yellow is considered as a positive signal.

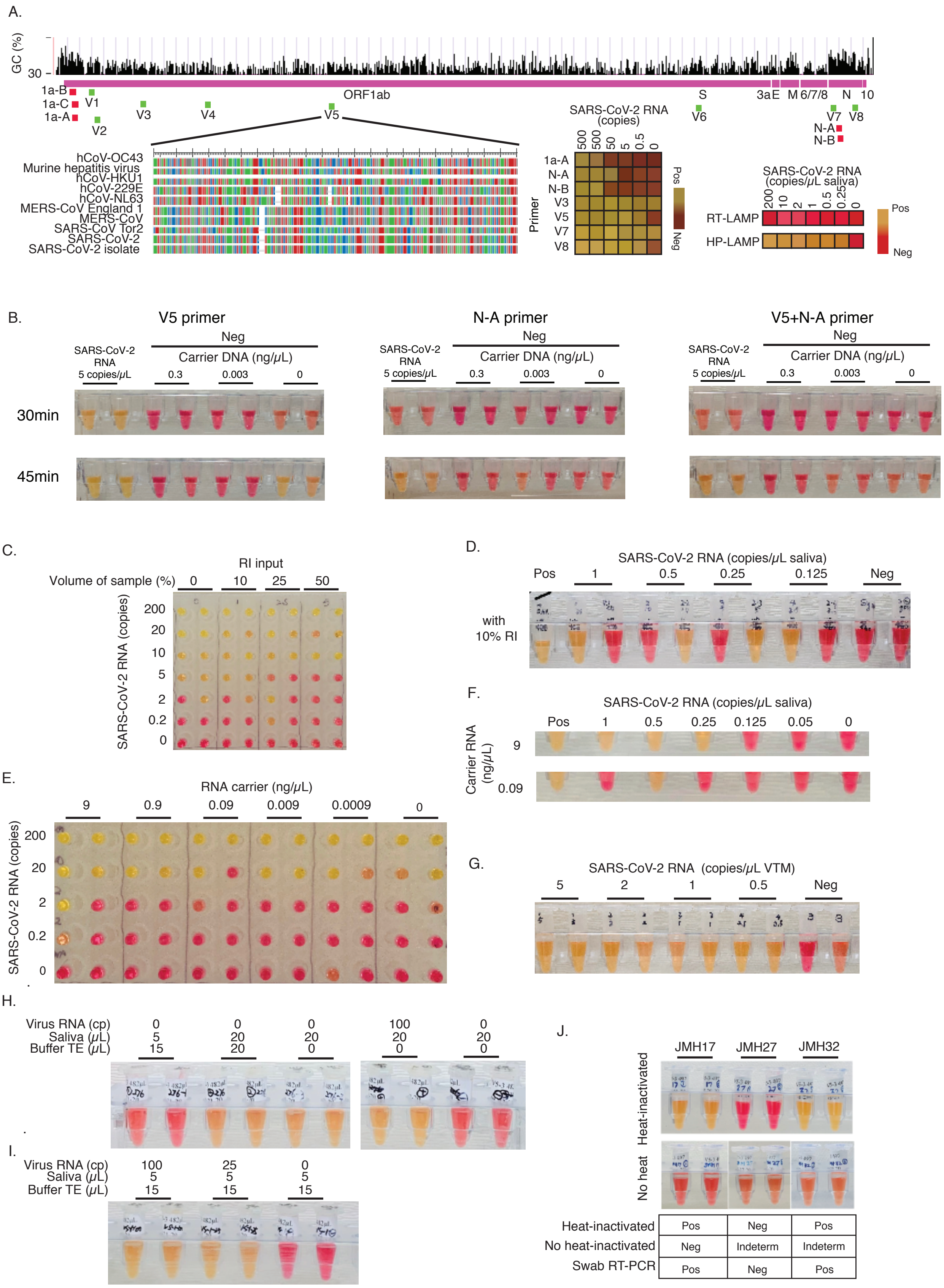
A.
GC (%)
30
1a-B
1a-C
1a-A
V1
V2
V3
V4
V5
ORF1ab
S
3a
E
M
6/7/8
N
10
V6
V7
V8
N-A
N-B
hCoV-OC43
Murine hepatitis virus
hCoV-HKU1
hCoV-229E
hCoV-NL63
MERS-CoV England 1
MERS-CoV
SARS-CoV Tor2
SARS-CoV-2
SARS-CoV-2 isolate
SARS-CoV-2 RNA (copies)
500 500 50 5 0.5 0
Primer
1a-A
N-A
N-B
V3
V5
V7
V8
Pos
Neg
SARS-CoV-2 RNA (copies/μL saliva)
200 10 2 1 0.5 0.25 0
RT-LAMP
HP-LAMP
Pos
Neg
B.
V5 primer
N-A primer
V5+N-A primer
Neg
SARS-CoV-2 RNA 5 copies/μL
Carrier DNA (ng/μL)
0.3 0.003 0
30min
45min
C.
RI input
Volume of sample (%)
0 10 25 50
SARS-CoV-2 RNA (copies)
200
20
10
5
2
0.2
0
D.
SARS-CoV-2 RNA (copies/μL saliva)
Pos 1 0.5 0.25 0.125 Neg
with 10% RI
E.
RNA carrier (ng/μL)
9 0.9 0.09 0.009 0.0009 0
SARS-CoV-2 RNA (copies)
200
20
2
0.2
0
F.
SARS-CoV-2 RNA (copies/μL saliva)
Pos 1 0.5 0.25 0.125 0.05 0
Carrier RNA (ng/μL)
9
0.09
G.
SARS-CoV-2 RNA (copies/μL VTM)
5 2 1 0.5 Neg
H.
Virus RNA (cp) 0 0 0 100 0
Saliva (μL) 5 20 20 20 20
Buffer TE (μL) 15 20 0 0 0
I.
Virus RNA (cp) 100 25 0
Saliva (μL) 5 5 5
Buffer TE (μL) 15 15 15
J.
JMH17 JMH27 JMH32
Heat-inactivated
No heat
Heat-inactivated Pos Neg Pos
No heat-inactivated Neg Indeterm Indeterm
Swab RT-PCR Pos Neg Pos

Fig. S1

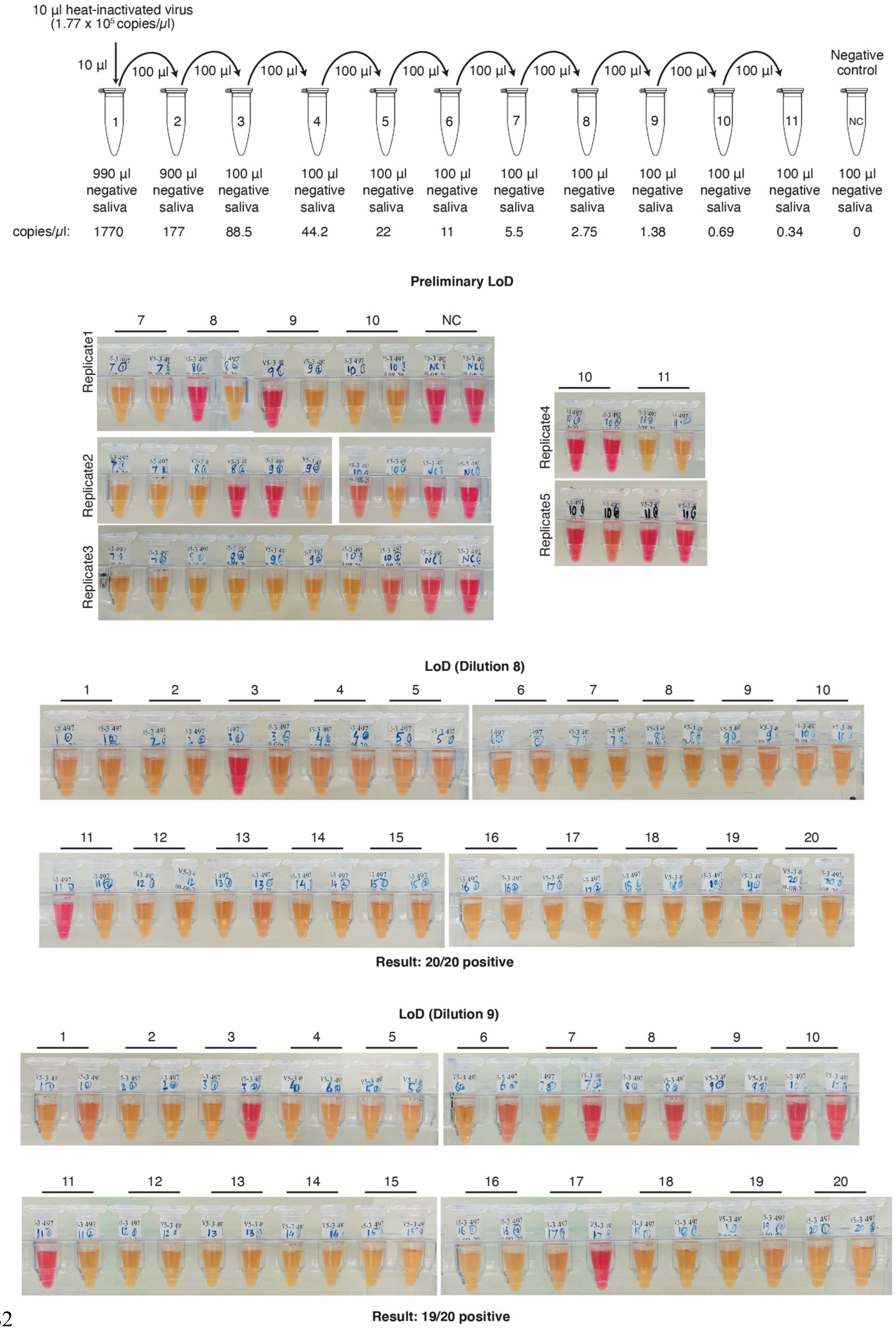
10 µl heat-inactivated virus
(1.77 x 10^5 copies/µl)
10 µl
100 µl
1
2
3
4
5
6
7
8
9
10
11
NC
Negative control
990 µl negative saliva
900 µl negative saliva
100 µl negative saliva
copies/µl:
1770
177
88.5
44.2
22
11
5.5
2.75
1.38
0.69
0.34
0
Preliminary LoD
Replicate1
Replicate2
Replicate3
Replicate4
Replicate5
LoD (Dilution 8)
Result: 20/20 positive
LoD (Dilution 9)
Result: 19/20 positive

Fig S2

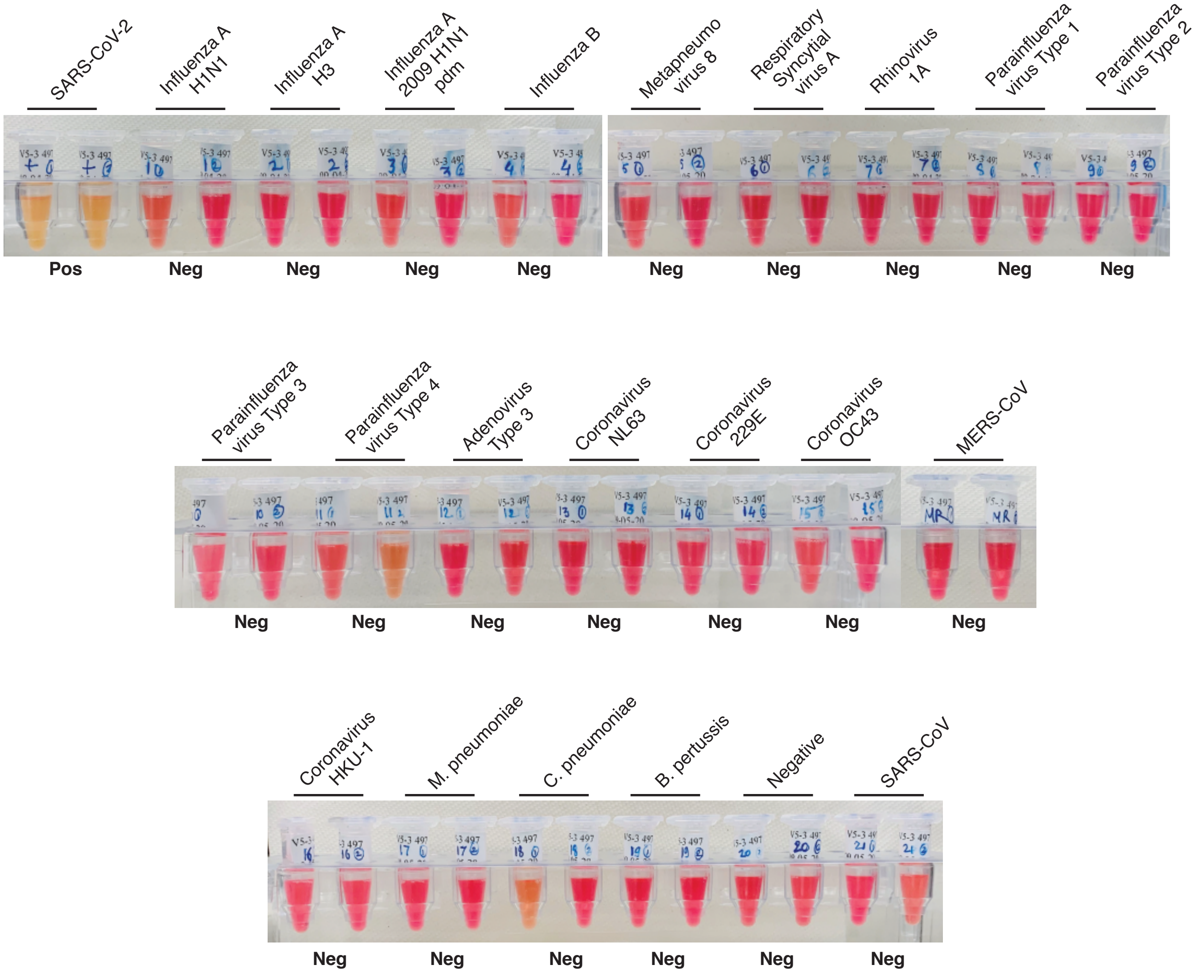

Figure S3. Cross-reactivity of HP-LAMP assay on common pathogens.
Inactivated known respiratory pathogens (n=21) along with inactivated SARS-CoV-2 virus were tested using HP-LAMP assay. All pathogens showed negative detection results in HP-LAMP assay, expect for SARS-CoV-2 virus.

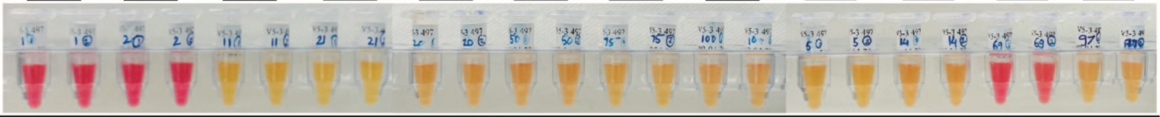

	JMH15	JMH12	JMH32	JMH10	JMH17	JMH42	JMH2	JMH4	JMH37	JMH43	JMH5	JMH7
HP-LAMP+ saliva results	Neg	Neg	Pos	Pos	Pos	Pos	Pos	Pos	Pos	Pos	Neg	Pos
NP swab RT-PCR results	Neg	Neg	Pos	Pos	Pos	Pos	Pos	Pos	Pos	Pos	Neg	Pos

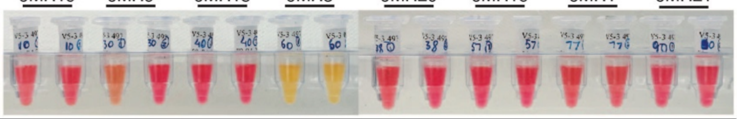

	JMH19	JMH9	JMH18	JMH3	JMH20	JMH16	JMH1	JMH21
HP-LAMP+ saliva results	Neg	Neg	Neg	Pos	Neg	Neg	Neg	Neg
NP swab RT-PCR results	Neg	Neg	Neg	Pos	Neg	Neg	Neg	Neg

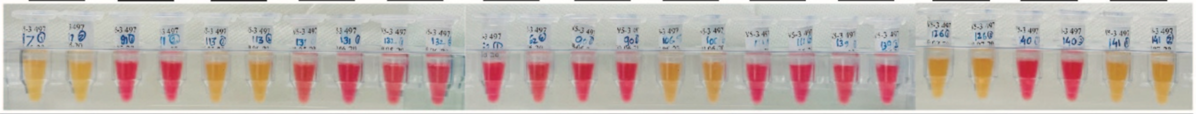

	JMH87	JMH91	JMH113	JMH131	JMH132	JMH62	JMH90	JMH106	JMH111	JMH139	JMH126	JMH140	JMH141
HP-LAMP+ saliva results	Pos	Neg	Pos	Neg	Neg	Neg	Neg	Pos	Neg	Neg	Pos	Neg	Pos
NP swab RT-PCR results	Pos	Neg	Pos	Neg	Neg	Neg	Neg	Pos	Neg	Pos	Pos	Neg	Pos

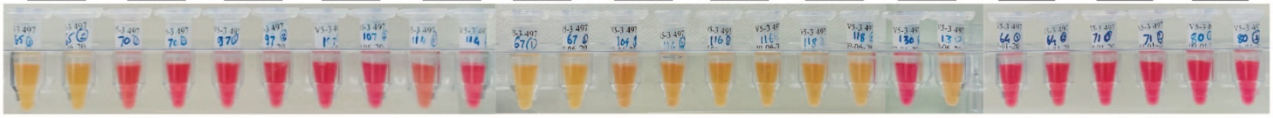

	JMH65	JMH70	JMH97	JMH107	JMH114	JMH67	JMH104	JMH116	JMH118	JMH130	JMH64	JMH71	JMH80
HP-LAMP+ saliva results	Pos	Neg	Neg	Neg	Neg	Pos	Pos	Pos	Pos	Pos	Neg	Neg	Neg
NP swab RT-PCR results	Pos	Neg	Neg	Neg	Neg	Pos	Pos	Pos	Pos	Pos	Neg	Neg	Neg

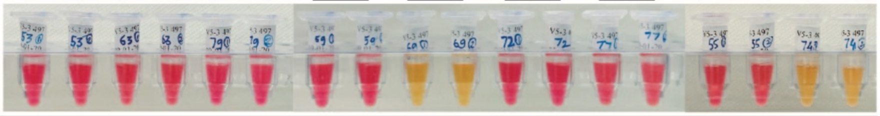

	JMH53	JMH63	JMH79	JMH59	JMH69	JMH72	JMH77	NYC93	NYC70
HP-LAMP+ saliva results	Neg	Neg	Neg	Neg	Pos	Neg	Neg	Neg	Pos
NP swab RT-PCR results	Neg	Neg	Neg	Neg	Pos	Neg	Neg	Neg	Pos

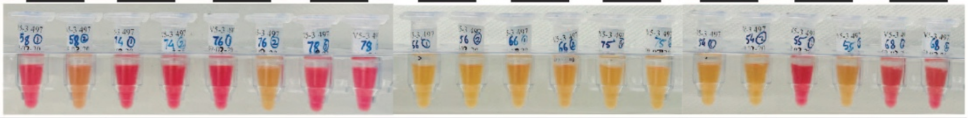

	JMH58	JMH74	JMH76	JMH78	JMH56	JMH66	JMH75	JMH54	JMH55	JMH68
HP-LAMP+ saliva results	Pos	Neg	Pos	Neg	Pos	Pos	Pos	Pos	Pos	Neg
NP swab RT-PCR results	Neg*	Neg	Pos	Neg	Pos	Pos	Pos	Pos	Pos	Neg

Figure S4. HP-LAMP testing results on clinical saliva samples.
Blinded clinical samples (n=65) were subjected to HP-LAMP assay, and the testing results were compared with paired RT-PCR results from nasopharyngeal swabs.

A.

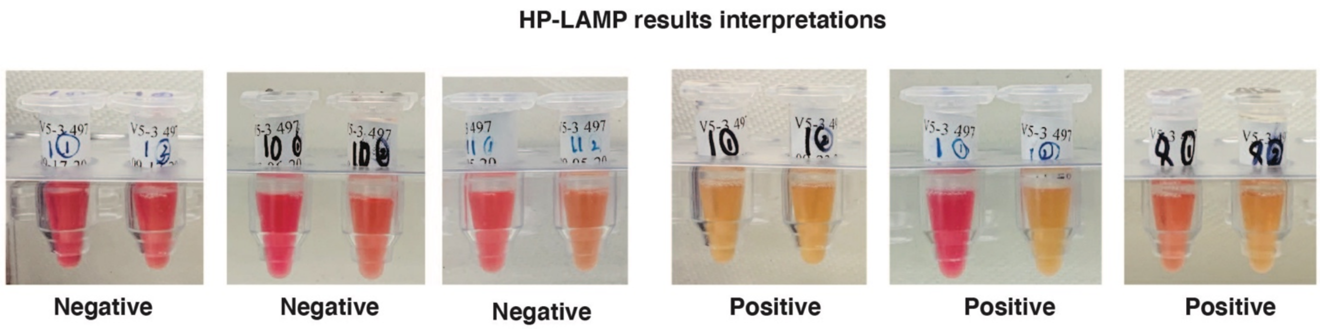

B.

Color	Interpretation
Yellow/Yellow	Positive signal
Red/Red	Negative signal
Yellow/Red	Positive signal
Yellow/Orange	Positive signal
Red/Orange	Negative signal
Orange/Orange	Negative signal

Figure S5. Interpretation of HP-LAMP colorimetric results. A) Representative HP-LAMP colorimetrc Results. Yellow: positive; Red: negative; orange: negative. HP-LAMP is tested in duplicate, at least 1 out of 2 tubes turned yellow is considered as a positive signal. **B). Table of color changes correlate with positivity.**
